# Impaired Learning From Errors and Punishments and Maladaptive Avoidance—General Mechanisms Underlying Self-Regulation Disorders?

**DOI:** 10.3389/fpsyt.2020.609874

**Published:** 2021-01-26

**Authors:** Marek Wypych, Marc N. Potenza

**Affiliations:** ^1^Laboratory of Brain Imaging, Nencki Institute of Experimental Biology of Polish Academy of Sciences, Warsaw, Poland; ^2^Department of Psychiatry, Yale University School of Medicine, New Haven, CT, United States; ^3^Child Study Center, Yale University School of Medicine, New Haven, CT, United States; ^4^Department of Neuroscience, Yale University, New Haven, CT, United States; ^5^Connecticut Mental Health Center, New Haven, CT, United States; ^6^Connecticut Council on Problem Gambling, Wethersfield, CT, United States

**Keywords:** self-control, impulsive behavior, punishment, emotional regulation, avoidance learning, substance-related disorders, procrastination, addictive behaviors

## Abstract

Self-regulation (SR) is an important human function that relates to quality of life in multiple domains including mental health. Previous studies have found important correlates of low SR including impulsivity and poor emotional regulation; however, underpinnings of low SR are incompletely understood. Individuals low in SR frequently engage in maladaptive behaviors (substance abuse, procrastination, etc.) despite negative consequences. This phenomenon suggests that impaired learning from errors and punishments may be important mechanisms underlying low SR. Consistently, previous studies observed impaired error processing in a wide spectrum of individuals with low SR and impaired learning from errors and punishments in SR-related disorders. We also note a possible role for poor emotional regulation and refer to concepts suggesting that engaging in maladaptive behaviors may serve as short term emotion regulation strategies aimed at avoiding or alleviating negative affect. We speculate on transdiagnostic factors underlying poor SR. We propose that impaired error processing (possibly related to striatal functioning) may prevent subjects with low SR from learning from errors and punishments and thus learning better SR skills or tendencies. Additionally, impaired coping in emotionally challenging situations, possibly related to prefrontal-cortical functioning, may lead to maladaptive avoidance. Moreover, maladaptive behaviors may be reinforced by the temporary decreases in negative affect and rewarding values of behaviors. Given existing knowledge gaps, we call for more extensive research and describe possible directions and challenges for future studies.

## Introduction

Self-regulation (SR) is an important human ability or tendency, with the former relating more to cognitive functioning and the latter more to behavioral traits. Levels of SR differ across individuals and correlate with quality of life in many domains including mental health (1). Thus, the topic is gaining attention among researchers in different fields of science. Previous studies have found multiple correlates of low SR including impulsivity and poor executive functioning and emotional regulation (2). Nonetheless, an incomplete understanding of underpinnings of low SR persists. Here we explore the notion that impaired learning from errors and punishments may underlie poor SR, and maladaptive avoidance may result from and perpetuate low SR.

People make many decisions regarding their actions, some of which are perceived at the time or later to be in error and may lead to negative consequences. For most people, such negative consequences serve as punishments and lead to learning and acting differently in similar future situations. However, people with low SR may frequently engage in maladaptive behaviors (e.g., substance abuse, procrastination, binge eating, gambling, etc.) despite negative consequences. Such data suggest that these individuals may have impaired mechanisms of learning from failures and punishments.

The topic of punishment and avoidance learning has arguably been receiving more attention recently (3, 4). While some human studies have focused on avoidance in relation to anxiety disorders (5) and relatively few experimental papers have investigated learning from punishments and/or avoidance learning in relation to broadly understood SR, some data suggest that subject groups low in SR may learn less from errors and punishment (6). These studies complement a more extensive behavioral and neuroimaging research in SR, impulsivity, substance use disorders and behavioral addictions (2, 7–12).

In the following sections, we first review studies suggesting error-processing may be impaired in a range of individuals with low SR. Next, we summarize data relating impaired error-processing to less effective learning from punishments. We then describe studies demonstrating impaired learning from errors and punishment in SR-related disorders and behaviors (in addictive disorders and procrastination). We also consider a role for poor emotional regulation and suggest that short-term avoidance of negative emotions may be a mechanism motivating people to engage in the maladaptive behaviors that may result in long-term negative consequences. Based on striatal and prefrontal systems, we propose a transdiagnostic model connecting impaired coping and learning from errors and punishments with tendencies to engage in maladaptive behaviors. Finally, we describe the need for more extensive research and suggest some possible future directions.

### What Is Self-Regulation?

Following (2), we consider SR in a broad sense as, “the intrinsic processes aimed at adjusting mental and physiological state adaptively to context.” SR “encompasses cognitive control, emotion regulation, and top-down and bottom-up processes that alter emotion, behavior, or cognition to attempt to enhance adaptation (…) strategic/deliberative as well as reactive/automatized processes and their reciprocal influences.” This broad definition encompasses also self-control defined as “top-down aspects of SR” (2). From a clinical perspective, individuals with low SR often engage in maladaptive behaviors like substance abuse, problematic gambling, procrastination, excessive gaming, binge eating and other behaviors, and they will be a focus of the present considerations.

While punishment, negative motivation and negative affect may have different underpinnings and associations, they may also share features relevant to SR. As such, we will consider these processes generally, while noting some unique psychological and neurobiological underpinnings.

## Error Processing

Error processing is often measured using specific tasks (e.g., Go/No-go, stop-signal or flanker) and assessment of error-related brain activity often employs EEG or fMRI approaches. Commission of an error results in activation of a network of brain regions including the anterior cingulate cortex (ACC); see (13) for a meta-analysis. Multiple studies of substance additions (involving cannabis, opioid, cocaine, or tobacco use) and behavioral addictions (involving gambling or gaming) suggest diminished error-related ACC activity measured with fMRI or as lower amplitude of error-related negativity (ERN) in EEG studies; see (14) for a meta-analysis. Interestingly, however, in people with alcohol dependence, increased ERN was found, possibly in relation to higher anxiety (15). Diminished error-related brain activity has also been found in people with criminal recidivism (16), procrastination (17, 18), and high impulsivity (19). These results suggest impaired error processing may link transdiagnostically to multiple groups with low SR.

Measurement of startle reactions after errors and correct trials suggests that errors are aversive (20). Amplitude of the ERN component related to degree of startle, consistent with a recent study in which assessments of error sensitivity (i.e., the fear of making mistakes) was correlated with ERN measures in children (21). These results suggest that errors may be less salient and arousing in individuals with lower ERNs. A study of impulsivity employing a flanker task with separated reward and punishment conditions found impulsive subjects to exhibit particularly low ERN components in punishment trials (22). Together these results suggest monitoring difficulties, especially in punishment contexts, and/or impaired processing of aversive values of errors and punishments in individuals with low SR.

Noteworthy errors can also be interpreted as conflicts between the assumed goals and the obtained situations [e.g., (23)], and conflicts have also been proposed to represent aversive signals (24). In light of the theory of expected value of control (25), the activity of ACC may be interpreted as a more general signal regulating cognitive control in demanding situations. This could suggest that individuals characterized with lower error-related ACC activity could also show cognitive control deficits in other situations. A recent review (26) has noted correlations between neural correlates of error processing (ERN amplitudes) and multiple measures of cognitive control. The measures, however, did not assess performance related to learning from errors. Taking the above-mentioned results together, one may hypothesize that lower ERNs and lower SR may concurrently relate to impaired learning from errors and punishments.

## Learning From Errors and Punishments

Individual differences exist relating to tendencies to learn from rewards or punishments. A probabilistic cognitive reinforcement learning task used in one study (27) had two stages: learning and inference. In the learning phase, participants were presented with three pairs of stimuli (AB, CD, EF, one pair at a time), were to choose one of the stimuli from a pair, and were provided with feedback. Choosing A resulted in reward in 80% of trials and in punishment with 20% probability, while stimulus B had the opposite contingency. In the CD pair, the probabilities were 70 and 30%, and in the EF pair they were 60 and 40%. During the learning phase, participants learned to choose A over B; however, to check whether they had learned to pursue rewards (choose A) or to avoid punishments (avoid B), a second phase of the task was conducted. During the inference phase, participants again were to choose one of two stimuli, but the stimuli were mixed in different pairs (i.e., AC, AD, AE, AF and BC, BD, BE, BF) and no feedback was provided. Analysis of performance from the inference stage provides insight into whether participants show biases toward learning from rewards (more frequent choices of A) or punishments (more frequent avoidance of B). The authors recorded EEG during the task and found that learning from punishments correlated with ERN amplitudes. Unfortunately, no self-regulation-related questionnaires were employed in the study. However, together with information presented above, the results suggest that subjects with low SR, and thus low ERNs, would show decreased learning from punishments. Several recent studies support this possibility.

An fMRI study employing a spatial paired-associate learning task suggests impaired learning from errors in people who use cannabis (28). In this study, participants were instructed to remember and recall associations of numbers with spatial locations on the screen. After a first round of recall, subjects were presented with the correct numbers, and, if they were unsuccessful, they could improve their performance in a second round of recall. The proportion of corrected errors was significantly lower in individuals who used cannabis vs. those who did not. Neuroimaging results revealed significantly lower error-related brain activity in several regions including the ACC in individuals who used cannabis. Moreover, the ACC was implicated in a group-by-error-type interaction (group: cannabis use vs. non-use; error type: corrected vs. repeated). The interaction suggested higher ACC activity in the non-using group during processing of errors that were later corrected. These results suggest impaired error processing and learning from errors in relation to cannabis use, although disentangling whether impaired learning from errors could have led to cannabis abuse or vice versa would require further studies.

A monetary version of this task, allowing manipulation of the quantity of the monetary outcomes and to separate rewards and punishments, was used to investigate cigarette smoking (29). The study revealed that non-smoking subjects learned better than smoking subjects from small rewards and large punishments. fMRI results showed no error-related group differences in the ACC. The smoking group, however, showed higher activity of the right dorsolateral prefrontal cortex (DLPFC) during recall and during re-encoding of errors corrected in the second round. The authors noted that a “greater need for attentional control, or reduced efficiency in translating DLPFC activation into attentional control” may be evident in individuals who smoke cigarettes.

Impaired learning from punishments has been suggested in opioid addiction during an acquired equivalence task (30). During the first phase of the experiment, subjects were to learn by trial and error associations between antecedent stimuli and consequences. Importantly, learning of associations of two of four antecedent stimuli was reward-based (positive feedback and points gained for correct choices and no feedback for incorrect choices) and learning of the other two was punishment-based (no feedback for correct choices and negative feedback and points lost for incorrect choices). Relative to healthy comparison subjects, opioid-addicted participants needed more trials and committed more errors to reach a desired outcome while learning from punishments. However, no significant group difference was found in reward-based learning. The authors concluded that a “selective deficit in learning from punishment could contribute to processes by which addicted individuals continue to pursue drug use even at the cost of negative consequences.”

We have conducted a behavioral study where students high and low in procrastination performed probabilistic reversal learning tasks with separate reward and punishment conditions (31). During the reward condition, participants were to repeatedly choose between two stimuli, where one had higher (75%) and the other had lower (25%) probabilities of monetary reward. Participants were instructed to maximize gains. From time to time, the stimulus-reward contingencies reversed (the previously better stimulus became worse and vice versa). In the punishment condition, the situation was analogous, and participants were instructed to minimize losses. Analysis was based on the Rescorla-Wagner model (32) as applied to human data (33). The model permits calculation of learning rates (i.e., rates of change of conviction about stimuli-reward contingencies) and exploitation-exploration balance of each participant in each condition (reward/punishment). The analysis revealed significantly lower learning rates in students high in procrastination regardless of condition. This group also demonstrated less exploration (or more persistence). A group-by-condition interaction indicated greater persistence in highly procrastinating subjects during the punishment condition. These results suggest that individuals high in procrastination are less flexible in learning than those low in procrastination, especially in punishment contexts.

Taken together, the above-mentioned results suggest impaired learning from errors and punishments across diagnostic boundaries in individuals low in SR.

## Maladaptive Avoidance and Impaired Emotional Regulation

The experiments described in the previous section confront subjects with choices and do not allow for or assess avoidance. Employing different experimental paradigms could assess avoidance behaviors that may better resemble real-life situations. Relatively few such studies have been conducted, with several described below.

In one study of heroin-dependent and non-dependent control subjects, participants were asked to perform an escape-avoidance task in the form of a computer game in which they controlled a spaceship with the goal of earning points by shooting enemy spaceships (34). During the first twelve “acquisition trials,” a warning signal announcing a bomb coming was periodically displayed for 5 s. Appearance of a bomb resulted in a reduction of points unless subjects hid their spaceship in a “safe box.” During the next twelve “extinction trials,” no bomb appeared after the warning signal. Women performed significantly worse on the task, and group differences were found only in male subjects. Heroin-dependent males scored significantly fewer points than control subjects because they spent significantly more time hiding in the safe box during the warning signal and a good while after the “bomb period,” during both acquisition and extinction trials. This study suggests that heroin-addicted vs. non-addicted males present exaggerated avoidance behavior that may result in reduced opportunities to obtain rewards.

A study employing the same task in alcohol-dependent and healthy men (35) led to similar and different results. Alcohol-dependent men escaped the bomb situation more often than healthy men. They also tended to spend more time hiding during the warning signal in the acquisition but not in the extinction trials. However, alcohol-dependent men scored more points in total as they shot significantly more enemy spaceships than healthy control men, especially during the extinction phase. The authors interpreted the results as, “supporting the idea that both positive and negative reinforcement are important components underlying addictive behaviors.”

Maladaptive avoiding may represent an important consideration in addictions. We use the term maladaptive avoidance as reflecting managing stressful situations by not addressing them directly, but by averting attention from them (c.f. “avoidance coping” in APA Dictionary) (36), and this may in turn eventually lead to negative consequences including more stress and negative emotions. Whether behaviors observed in the above-mentioned experimental studies are suitably modeling real-life maladaptive avoidance may be subject to interpretation and further investigation; however, a role for maladaptive avoidance in individuals characterized by poor SR (including those with addictions) was proposed decades ago (37, 38). In related models, engaging in maladapitve or addictive behaviors (e.g., substance abuse, gambling, proctrastination, binge eating) may serve as a short-term emotion-regulation strategy aimed at avoiding or alleviating negative affect [consistent with negative reinforcement motivations and self-medication models of addictions (39), among others (8, 40)]. Previous studies using self-report measures support the notion of engaging in potentially addictive behaviors like substance use (41), gambling (42), procrastination (43), and internet use and gaming (44, 45) for avoiding or alleviating negative mood states.

The described preference to obtain short-term rewards or relief over avoiding possibly larger long-term punishments in people with low SR may also may be interpreted in terms of steep delay discounting or a tendency to value immediate future events higher than more distant ones. Indeed, a recent meta-analysis of delay discounting in addictive behaviors found a small but highly significant correlation (46). Interestingly, delay discounting was correlated with procrastination when measured with questionnaires (47, 48), but not when measured with tasks (49). Most previous studies focused however on delayed reward discounting, and did not include punishments. Future studies should therefore examine delayed punishment discounting, and also consider whether there may be causal relationships between delay discounting and maladaptive avoidance.

The above-mentioned results suggest that maladaptive avoidance may represent a mechanism involved in disorders characterized by low SR. Maladaptive avoidance tendencies may reflect strategies developed to compensate for poor coping in response to challenging situations (for example, to decrease stress when exams approach, students could play computer games instead of studying). Alternatively, both maladaptive avoidance and impaired learning from errors and punishments may indicate impaired cognitive and behavioral control during emotionally challenging situations in which negative emotions and/or threat of punishment are experienced or anticipated.

## Discussion and Possible Mechanisms

SR failures related to negative affect may result from imbalances between subcortical and frontal regions; e.g., insufficient frontal top-down control “either due to particularly strong impulses or when prefrontal function itself is impaired” (50). Indeed, both lack of reward and receipt of punishment have led to activation of the right DLPFC in healthy subjects (51). These findings suggest increased behavioral control in such situations. According to the theory of expected value of control (25), cognitive control is implemented by the lateral PFC in response to ACC signaling a potential need for it. Therefore, it may be predicted that activity of these prefrontal brain regions related to punishment or negative affect could differ in individuals high and low in SR. Indeed, abstinent cocaine-dependent participants showed hypoactivity of the ACC and right DLPFC in relation to punished errors in the Go/No-go task (52). We have found similar results (diminished ACC and right DLPFC activity during punishment condition in the Go/No-go task) in individuals high in procrastination (17). These findings are in line with prior theories (50) and suggest that impairment of prefrontal control by punishment threat or negative emotions may operate for many subject groups characterized by low SR. Note, however, that the previously mentioned study (29) showed higher DLPFC activity in tobacco-smoking individuals during re-encoding of later-corrected errors, possibly reflecting higher difficulty and/or effort needed to correctly perform in the task, or impaired mechanisms of regulation of intensity of implemented control [c.f. (25)].

Prefrontal-striatal mechanisms linked to SR may be amenable to interventions, with transdiagnostic implications. Both regulation of emotion and regulation of motivation (e.g., craving in addictive disorders) have been implicated in groups characterized by low SR. Among individuals who smoke tobacco, regulation of craving has been found to involve prefrontal control over striatal cue responsiveness (53). Prefrontal regions (PFC, ACC) and circuitry (a fronto-cingulo-parietal network) were less activated and engaged, respectively, in individuals with internet gaming disorder vs. those without during emotional regulation (54). Recently, both impaired regulation over responses to primary rewards and addictive cues have been reported in internet gaming disorder (55), and stimulation of the right DLPFC enhanced both regulation of craving and negative emotions in individuals with internet gaming disorder (56). Taken together and in line with prior theories of internet gaming disorder (57) and other addictive behaviors (8, 58), these findings suggest that interventions targeting increased prefrontal control over subcortical emotions/motivations may help multiple groups with low SR.

Concerning the balance between subcortical and frontal regions, dysregulation of dopaminergic circuits may be one mechanism underpinning impaired reward, punishment, and error processing, and thus underlie impulsivity, addictions and possibly low SR in general (59–62), although dopamine may play a more central role in some disorders than in others (63–65). At least two non-exclusive dopamine-related mechanisms may underlie aspects of low SR.

First, a “No-Go” pathway could be deficiently functioning in low SR as has been proposed as one element in some models of addiction (66) and could reflect higher striatal and lower prefrontal activity. However, addictive behaviors have also been linked to lower striatal activation [e.g., in reward anticipation in substance use and gambling disorders (67), with similar findings in binge eating disorder (68, 69) and internet gaming disorder (70)]. In several of these studies, blunted striatal activation was linked to increased impulsivity (71–73), suggesting a relationship to SR. These findings are in line with reward deficiency models of addiction (74) and suggest more complex etiologies relating to SR in addictive disorders. Blunted ventral striatal activation during a prospect phase of reward and loss processing has been linked to disadvantageous decision-making in people with and without gambling disorder, suggesting another route by which reward and loss processing may link to SR impairment (75). Although some models suggest that striatal dopamine may underlie aspects of human addictions (76), findings may be strongest for stimulant use disorders (63), and even in such disorders, dopamine receptor availability may show differential relationships with measures of disease severity (77).

When considering the involvement of the striatum in SR, one should be mindful of complexities involving regions of the striatum and how they may relate to SR, including changes within individuals over time both developmentally and from life experiences including psychopathology (78, 79). For example, data suggest that the ventral striatum may be more linked to impulsive behaviors and dorsal striatum to more habitual behaviors, with these regions components of different parallel cortico-striato-pallidal-thalamo-cortical circuits that are involved in different stages of disorders (e.g., addictions, impulse control disorders) characterized by impairments in SR (79–84). Given these data, a shift from more ventral to dorsal striatal involvement has been proposed for addictions as they become more instantiated (84). More recently, a study of healthy adults performing a naturalistic maze-navigation task identified functionally segregated regions of the ventral striatum that separately encode specific aspects of performance [effort activation, movement initiation and effort discounting of rewards (85)]. Furthermore, opposing patterns of activation related to effort activation and discounting were associated with striatal encoding of effort during effort-based decision-making. The authors suggested that the dorsomedial region of the striatum that has been previously understood as being linked to action may rather be involved in assessing cost of effort, raising questions regarding prior interpretations of striatal “reward” signals. Taken together, the findings indicate that more research is needed to examine involvement of specific striatal regions and circuits in studies of the neurochemical and neurocircuitry underpinnings of SR. Furthermore, additional circuitry should be considered, consistent with recent models of addiction (8, 40).

Second, considering the diminished error-related ACC activity, it could be hypothesized that the errors are less salient in low SR individuals and thus impede learning on errors. Early data (86) suggested that error-related ACC activity influenced learning not to repeat erroneous behaviors, and this may in part reflect dopamine-dependent reinforcement signals from the basal ganglia. This view was supported by data from patients with Parkinson's disease participating in a probabilistic reinforcement learning task (87) similar to the one described above (27). Unmedicated Parkinson's patients, considered characterized by low dopamine, learned better based on punishments than on rewards; application of dopamine-replacement medications reversed this pattern, suggesting that lower levels of dopamine may support D2-like-receptor-dependent “No-Go” learning based on errors and punishments, while high levels of dopamine may shift the balance toward D1-like-receptor-related “Go” behaviors (87). This interpretation is consistent with results of correlations between ERN amplitudes and learning from punishments in healthy subjects (27). This leads to speculation that people with lower ERNs and lower tendencies to learn from punishments may be characterized by higher levels of striatal dopamine, consistent with findings suggesting positive correlation between impulsivity and striatal dopamine measures (88, 89). Buckholtz and colleagues suggested that high levels of striatal dopamine in impulsive subjects may result from lower levels of D2/D3 autoreceptors in the substantia nigra and ventral tegmental area leading to stronger dopamine cell activity and enhanced release of dopamine which could in turn increase impulsivity and promote “Go” behaviors (88). However, given inconsistent findings and complications of synthesizing information across studies [e.g., from Parkinson and non-Parkinson populations that may show important dopamine-related differences given the pathophysiology of the disorder and other factors (90, 91)], additional research is necessary.

**Figure 1:**
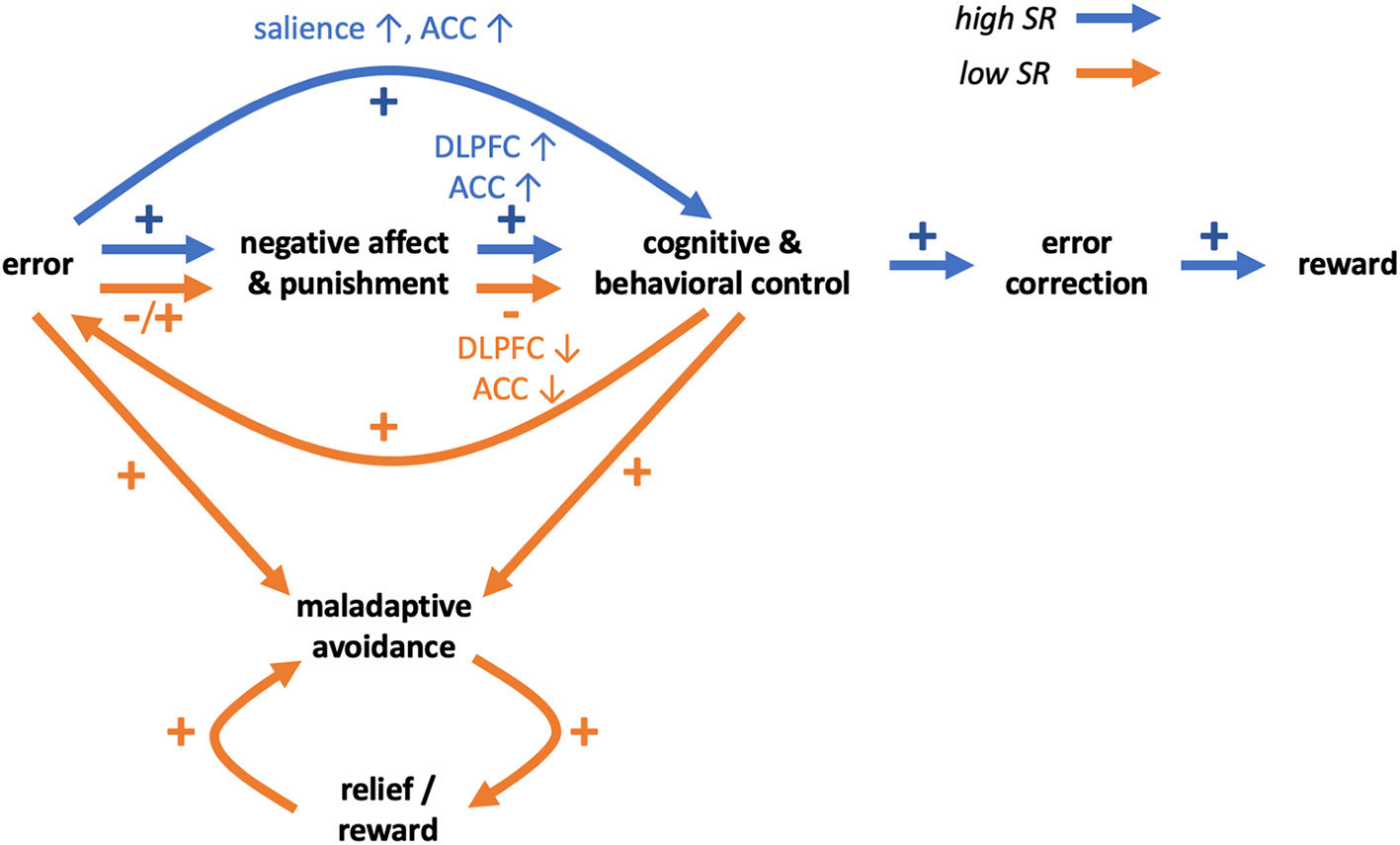
Schematic hypothetical transdiagnostic model of self-regulation behaviors and disorders in relation to impaired coping and learning from errors and punishments. Differences between high SR (blue arrows) and low SR (orange arrows) individuals may involve brain reactions to negative affect and punishments. In individuals high in SR, negative affect and punishments may lead to increased activity of prefrontal control regions (ACC, DLPFC) and increased cognitive and behavioral control. In individuals low in SR, ACC and DLPFC activity in emotionally challenging situations may be relatively lower and result in impairment of cognitive and behavioral control. In individuals high in SR, errors may be processed as more salient events and lead to increased monitoring and control, supporting correction of errors in the future, leading to more rewards and learning of self-regulation. In individuals low in SR, learning from errors and punishments may be impaired. Errors may be processed as less salient and aversive events and not result in increased monitoring and control, potentially leading to repeating of errors. Eventually however errors may result in punishments and negative affect and further decline of cognitive and behavioral control. Both because of error-processing and diminished control, individuals low in SR may preferentially engage in maladaptive behaviors. The behavioral pattern may be reinforced by the relief and rewarding value of substitute behaviors, eventually preventing individuals low in SR from learning self-regulation.

Summing up, in emotionally challenging situations prefrontal activity in individuals with low SR may be insufficient to effectively regulate emotional and motivational drives involving the striatum and other regions, and this may lead to difficulties in coping with negative situations. Additionally, individuals with low SR may not learn from errors and punishments, and this may relate to differences or impediments in error processing. Together, these mechanisms may impede coping in negative situations, hinder learning from such situations and impair learning SR (Figure 1).

Such maladaptive behaviors may be reinforced over time. In an fMRI study employing a probabilistic reinforcement learning task with separated rewards and punishments (51), the authors showed that both obtaining rewards and avoiding punishments lead to activation of the medial orbitofrontal cortex (mOFC). Given that the mOFC is a component of the ventromedial PFC that has been implicated in processing rewarding outcomes (92), the findings suggest that avoidance of aversive outcome may be rewarding. Similar conclusions were made in a different study employing a repeated acquisition approach-avoidance learning task and showed, among other regions, activations in anterior and posterior cingulate and ventral striatum both for rewards and avoidance of aversive outcomes (93). These studies showed that reward and avoidance learning rely largely on common brain networks, consistent with meta-analytic findings (94). Therefore, negative reinforcement (i.e., avoidance of punishment) may lead to consolidation of avoidance behaviors including maladaptive avoidance. Moreover, engaging in maladapitve behaviors (e.g., substance abuse, gambling, gaming) may be rewarding in and of themselves and thus additionally reinforce such behavioral patterns (Figure 1).

### Summary and Future Directions

In this manuscript, we discuss possible transdiagnostic mechanisms underlying poor SR. Impaired coping in emotionally challenging situations, related to decreased prefrontal activity, may promote avoidance of such situations in individuals with low SR. Moreover, impaired error processing may prevent individuals with low SR from learning from errors and punishments and impede effective correction of faulty behaviors and learning of SR itself. Additionally, maladaptive behaviors may be reinforced by short-term decreases in negative affect and by rewarding values of behaviors, leading to more persistent engagement (Figure 1).

Whether the model is valid and is transdiagnostic should be a subject of future studies, which may involve several challenges. First, regarding learning from errors and punishments, future studies may benefit from novel tasks (ideally applicable in neuroimaging studies) as many probabilistic reinforcement learning tasks often do not show between-group differences in task performance and require modeling approaches to show differences in learning strategies. Second, new experimental tasks, better mimicking real-life situations, should be developed to address learning of avoidance. Ideally, such tasks should allow participants a choice to avoid engagement in aspects of the task. They should also allow for distinguishing between learning of adaptive and maladaptive avoidance. Such human avoidance research tasks may encounter challenges that should be considered (95). Future studies should also check whether punishments, punishing contexts and negative affects evoked in different ways may lead to similar or different neuronal and behavioral effects. Future research could also refer to the theory of expected value of control and test which of the proposed components of cognitive control may be particularly impaired in individuals low in SR: estimation of expected outcomes, regulation of intensity of control, or monitoring of performance (25). Ideally, future research will address proposed mechanisms in a broad range of disorders characterized by low SR including substance use disorders, behavioral addictions, binge eating disorder, and other conditions, as well as in other relevant behavioral dimensions including procrastination and high impulsivity.

We believe that research addressing learning from negative consequences and coping in negative situations in disorders characterized by low SR may eventually contribute to the improvement of existing and/or development of new therapeutic approaches. Psychological interventions could possibly address emotional regulation, working with internal conflicts and/or training inhibitory control and error-awareness. Pharmacological therapies could aim at the regulation of function of neurochemical systems underlying motivational drives and cognitive control. Neuromodulatory approaches may be well-suited to increase prefrontal control over subcortical drives, particularly given the ability to target prefrontal cortical regions using transcranial direct current stimulation and repetitive transcranial magnetic stimulation.

### Limitations

Our proposed model should be considered cautiously. The understanding of individual differences, especially concerning SR, in processes underlying learning based on errors and punishment and avoidance learning is still developing; therefore, the model may currently be considered speculative. Existing data do not typically permit dissection of causes from consequences (e.g., of substance abuse); nevertheless, we believe the model is relevant to SR impairments. In considering a transdiagnostic mechanism underlying low SR, we considered together many disorders and behaviors characterized by low SR. In doing so, the model does not incorporate all mechanisms that may contribute to low SR, for example delay discounting or motivational processes, neither does it include factors relating, for example, to effects of specific substances. Noteworthy, we relate the results of the reported studies to learning of SR, repetitive engagement in maladaptive behaviors, and their long-term negative consequences, while the studies investigated almost exclusively short-term processes. Thus, it is imaginable that the experimental situations do not fully reflect real-life processes, especially with respect to preferring short-term rewards over avoiding of possibly larger long-term punishments, and further research in this area would likely improve understanding of mechanisms and help to refine the model. We also simplified and limited brain considerations largely to the striatum and PFC, and we largely did not discuss roles for particular subregions and other parts of the brain. We also did not discuss the broader range of neurochemical/neurotransmitter systems that may contribute to SR. However, we believe that these simplifications permitted proposing a model for how impaired coping under negative affect and impaired learning from errors and punishments may operate as transdiagnostic mechanisms underlying poor SR. We hope that future studies will test the proposed model, lead to the development of more detailed models and facilitate clinical advances related to poor SR.

## Data Availability Statement

The original contributions presented in the study are included in the article/supplementary material, further inquiries can be directed to the corresponding author/s.

## Author Contributions

MW conceptualized the manuscript. Both authors reviewed literature, wrote, and approved the submitted version of the manuscript.

## Conflict of Interest

MP has consulted for and advised Game Day Data, the Addiction Policy Forum, AXA, Idorsia, and Opiant/Lakelight Therapeutics; received research support from the Veteran's Administration, Mohegan Sun Casino, and the National Center for Responsible Gaming (no the International Center for Responsible Gambling); participated in surveys, mailings, or telephone consultations related to drug addiction, impulse-control disorders or other health topics; consulted for law offices and the federal public defender's office in issues related to impulse-control and addictive disorders; provided clinical care in the Connecticut Department of Mental Health and Addiction Services Problem Gambling Services Program; performed grant reviews for the National Institutes of Health and other agencies; edited journals and journal sections; given academic lectures in grand rounds, CME events and other clinical/scientific venues; and generated books or chapters for publishers of mental health texts. The views presented in this manuscript represent those of the authors. The remaining author declares that the research was conducted in the absence of any commercial or financial relationships that could be construed as a potential conflict of interest.
